# Perioperative Management of Metastatic Paraganglioma-Pheochromocytoma of the Humerus with the Aid of Regional Anesthesia

**DOI:** 10.1155/2020/2482793

**Published:** 2020-12-11

**Authors:** Iyabo O. Muse, Kumar Vivek, Noah A Bloomgarden, Amit Singla, David S. Geller

**Affiliations:** ^1^Department of Anesthesiology, Montefiore Medical Center, Albert Einstein College of Medicine, Bronx, New York, USA; ^2^Department of Medicine, Montefiore Medical Center, Albert Einstein College of Medicine, Bronx, New York, USA; ^3^Department of Orthopedic Surgery, Montefiore Medical Center, Albert Einstein College of Medicine, Bronx, New York, USA

## Abstract

A 38-year-old female with a past history of pheochromocytoma and subsequent malignant paraganglioma presented with right arm pain after a fall. Imaging demonstrated a malunited humeral shaft associated with a large cortical destructive lesion and extraosseous extension. Here, we report the use of a multidisciplinary team approach including an endocrinologist, anesthesiologist, and orthopedic surgeon in the perioperative management of a patient with metastatic paraganglioma undergoing a surgical resection of the humerus, internal fixation, reconstruction, and placement of endoprosthesis. The challenges of perioperative anesthetic management and the use of regional anesthesia, especially peripheral nerve block for perioperative pain management, are highlighted.

## 1. Introduction

Paragangliomas originate in the peripheral nervous system and are relatively rare tumors, with an incidence of approximately 1-2 per million people each year. Paragangliomas secrete catecholamines such as epinephrine and norepinephrine causing hypertension, tachycardia, and increase in the body's metabolic rate [1]. Unlike pheochromocytomas, which originate in the adrenal glands and produce similar catecholamines, paragangliomas are known as extra-adrenal pheochromocytomas. Paragangliomas are more likely to be malignant as high as 40–50% in some studies as compared with adrenal pheochromocytomas [2].

In this case, pathology revealed a malignant type of paraganglioma with lesions in the patient's humerus, tibia, 7th rib, and cervical spine. Through genetic testing, the endocrinologist diagnosed the patient with hereditary paraganglioma-pheochromocytoma associated with mutation in *SDHD* gene. People with hereditary paraganglioma-pheochromocytoma are typically diagnosed in their 30s and sometimes develop one or more paragangliomas that may also include pheochromocytomas [3]. The surgical resection of paragangliomas is challenging from an anesthetic perspective owing to the wild cyclic fluctuation in blood pressure seen in cases under general anesthesia. In this case, we report the value of adding regional anesthesia with general anesthesia in tapering volatile blood pressure fluctuation by blocking both motor and sensory receptors, thus reducing volatile agents and opioid utilization during surgery.

A HIPAA authorization was obtained from the patient for publication of this case report.

## 2. Case Description

A 38-year-old female with an extensive medical and surgical history presented to the orthopedic oncologist with a complaint of increasing right arm pain and an existing and worsening deformity after a fall sustained one year prior. Imaging studies revealed a malunited humeral shaft and an associated destructive lytic lesion (8.1 cm in length) of the proximal right humeral shaft with cortical destruction and extraosseous extension with close proximity to the brachial vessels. The patient had a prior history of hypertensive emergencies and was therefore on dual antihypertensive therapy (metoprolol and prazosin).

Her extensive surgical history dated back twenty years when she underwent a partial bladder wall and right adrenal resection for malignant paraganglioma confirmed by histopathology. Six years later, she fell and was found to have multiple lesions in her right tibia, left 7th rib, lower cervical spine, and right humerus on CT scan. A bone biopsy revealed metastatic paraganglioma. She was treated with systemic chemotherapy and underwent in remission for 10 years. Then, she sustained an injury affecting her back and her right shoulder. An MRI imaging raised concerns of tumor recurrence within her humerus with no recurrence in the adrenal gland. Laboratory testing revealed increased free normetanephrines (5410; ref </ = 148) and chromogranin A (782; ref 25–140 ng/ml) but normal free metanephrines and calcitonin.

Due to large tumor size (7.4 × 4.1 × 3.4 cm) and patient's right arm pain, a wide resection and reconstruction with a cemented intercalary endoprosthesis was planned. A multidisciplinary approach for perioperative management of the patient was initiated. The patient was admitted four days prior to surgery for medical optimization. On admission, patient's blood pressure was 152/82 mmHg, HR 73 bpm, and respiratory rate was 17. Blood pressure during the four days ranged (113–220)/(68–110) mmHg. Presurgery hospital medications included PO phenoxybenzamine 30 mg BID prior to surgery, metoprolol 75 mg BID, IV fluids 200 ml/hr, oxycodone 5 mg/10 mg, and lorazepam 2 mg PO once as needed. Twenty-four hours prior to surgery the patient's blood pressure and heart rate ranges were (113–148)/(68–94) mmHg and (65–98) bpm, respectively. Echocardiogram showed normal ejection fraction and no evidence of pulmonary hypertension. EKG was normal with a HR of 62 bpm.

**Figure 1:**
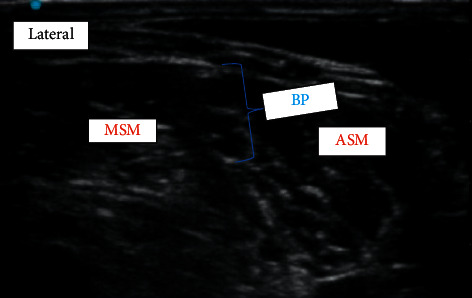
Image of interscalene brachial plexus (BP). The BP is seen positioned between the anterior scalene muscle (ASM) and the middle scalene muscle (MSM). In this particular image, the white arrow represents the needle location for local anesthetic injection.

On the morning of surgery, the patient was given midazolam 2 mg IV in the preoperative holding area. In the operating room, an arterial line was placed. After administering 100 mcg of IV fentanyl, an ultrasound-guided right interscalene brachial plexus nerve block was performed. A catheter was concomitantly inserted in-plane using a high frequency linear transducer with a 13-6 MHz bandwidth (Figure 1). A total of 12 ml of 0.5% ropivacaine was given for intraoperative pain, with increase in arm temperature and dilation of some hand veins noted prior to intubation. For the surgical procedure, general anesthesia with endotracheal intubation was induced with IV lidocaine (60 mg), etomidate (10 mg), propofol (50 mg), fentanyl (100 mcg), and rocuronium (40 mg). After induction, a central venous triple lumen catheter was inserted.

During the surgery, there were expected fluctuations in the blood pressure, with a range of (75–179)/(40–90) mmHg. The highest blood pressure was during surgical manipulation of the tumor, and the lowest blood pressure was after extraction of the mass from the surgical bed. Periods of hypotension were managed with titration of vasopressin 2-3units/hr and norepinephrine 5–10 mcg/min as well as the addition of fluid boluses. At the end of the procedure, the patient's blood pressure stabilized without requiring vasoactive support. She was extubated and transported to PACU with a BP of 110/52 mmHg. Surgical time was 4.5 hours. Total intake was 2.5 L Plasmalyte, 1L 5% albumin, and 1U PRBC (300 ml), and output was estimated blood loss of 1100 ml and urine output of 1300 ml.

Postoperatively, patient's blood pressure range was (110–118)/(52–58) mmHg and her HR was (86–91) bpm. The interscalene catheter infusion was started with 0.2% ropivacaine at 5 ml/hr for postoperative pain management. Pain scores and total opioid consumption during hospitalization (Table 1) showed a maximum pain score of 4 on a VAS scale of (0–10) in the first 24 hours after surgery. The interscalene catheter was removed on POD#2, and the patient was placed on oral opioids (oxycodone 5 mg/10 mg q4hrs prn), acetaminophen 975 mg q8hrs, and lidocaine 5% patch. Following the procedure, the patient did not require alpha blocker agents and was discharged home on POD#4 with oxycodone 5 mg q4hrs prn and lorazepam 2 mg orally daily as needed for anxiety.

**Figure 2:**
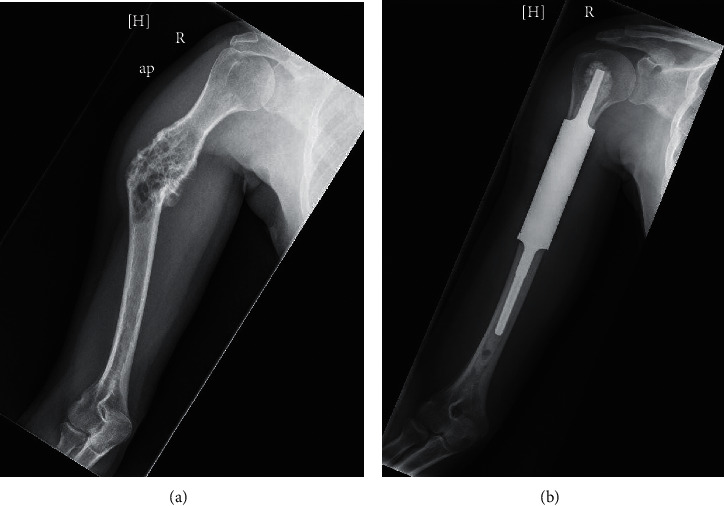
(A) Preoperative radiograph showing lytic lesion in the humerus shaft with deformity. (b) Postoperative radiograph showing well fixed intercalary humerus prosthesis.

Two weeks after surgery, she was seen in follow-up by both the orthopedic surgeon and the endocrinologist. The patient exhibited full range of motion of her right elbow and fingers and was neurovascularly intact (Figure 2). Repeat laboratory tests revealed a decrease in the total catecholamine level to 616pg/ml, a decrease in free normetanephrine to 188pg/ml, a normal metanephrines of 51pg/ml, and a normal chromogranin A of 128 ng/ml.

## 3. Discussion

Perioperative management of metastatic paraganglioma-pheochromocytoma is challenging for a number of reasons, not the least of which is the wild cyclic fluctuations of blood pressures due to excessive catecholamine secretion. In the more severe cases, this fluctuation can lead to end-organ damage such as cardiomyopathy and renal injury [4]. Since blood pressure and heart rate are exacerbated by pain, it is prudent to minimize perioperative pain using multimodal analgesia and regional anesthesia. It is also valuable to develop and agree upon a treatment plan prior to surgery using a multidisciplinary team. In this case, the treatment plan included early inpatient admission, pharmacologic alpha- and beta-adrenergic blockade, IV hydration, and regional anesthesia to achieve reasonable hemodynamics. A large bore IV, central venous catheter with or without a pulmonary artery catheter to guide fluid, and inotropic/vasoactive medication administration are also advised. Short acting vasodilators such as nitroglycerine and nitroprusside and short acting beta-1 selective antagonist such as esmolol and calcium channel blockers (nicardipine) are usually needed for hemodynamic optimization during tumor manipulation [5]. Once the tumor is removed, the patient may demonstrate profound hypotension due to the sudden withdrawal of baseline catecholamines. To combat hypotension, intravascular volume expansion and vasopressor agents are required to maintain the patient's hemodynamics. This choice is guided by the nature and duration of the patient's preoperative alpha-adrenergic blockade strategy. Use of vasopressin, which does not rely on alpha-adrenergic agonism, is beneficial to increase vasomotor tone [6].

Perioperative pain management is an important factor in controlling blood pressure. Peripheral nerve blocks provide excellent analgesia for hours to days (single shot or catheter) in patients undergoing painful surgical procedures. In this case, we performed an interscalene brachial plexus block with catheter placement to aid in perioperative pain management. This helped reducing the frequency of blood pressure fluctuation and also the amount of intraoperative opioid used during surgery, limiting it to 25 mcg of fentanyl. Postoperatively, the patient required opioid administration only after removal of the catheter on the second postoperative day.

Regional anesthesia with sedation could have alternatively been used as the primary anesthetic in lieu of general anesthesia. The interscalene nerve block provides both a motor and sensory blockade thus providing effective anesthesia and analgesia. However, in this case, the high risk of significant intraoperative bleeding due to the large tumor size and its vicinity to the humeral circumflex artery and the deep brachial artery led to the decision to use general anesthesia as the primary anesthetic.

This case outlines a number of important challenges including those posed by the surgery itself and those related to hemodynamic fluctuations. Having a carefully outlined preoperative plan with which to manage the swings in blood pressures, heart rate, and intravascular volume is critical to avoiding perioperative cardiopulmonary complications. A peripheral nerve block is a great option for limiting intraoperative management of swings in blood pressure due to surgical pain. In this case, the regional anesthesia technique aided in both an optimal intraoperative and postoperative outcome for the patient. The planning and preparation by our multidisciplinary team (orthopedic surgeon, anesthesiologists, and endocrinologist) allowed for a patient-centered approach and led to a smooth hospital course as well.

## Figures and Tables

**Table 1:** Postoperative pain scores (VAS, visual analog score) and opioid consumption in morphine equivalent.

Postoperative day (POD)	VAS score median (minimum-maximum)	Opioid consumption in oral morphine equivalent (mg)
POD#0	2 (0–4)	10
POD#1	0 (0–4)	24
^‡^POD#2	1 (0–3)	30
POD#3	4 (2–6)	33
POD#4	3 (1–3)	20
^*∗*^POD#5	1	5

^‡^Peripheral nerve catheter removed; ^*∗*^day of discharge.
